# The ash concentration of co-PDC clouds: implications for operational modelling and the aviation hazard

**DOI:** 10.1038/s44304-026-00214-7

**Published:** 2026-05-07

**Authors:** Marie Hagenbourger, Thomas J. Jones, Frances M. Beckett, Samantha L. Engwell

**Affiliations:** 1 Lancaster Environment Centre, Lancaster University, Lancaster, UK; 2 Met Office, Exeter, UK; 3 British Geological Survey, The Lyell Centre, Edinburgh, UK

**Keywords:** Climate sciences, Environmental sciences, Natural hazards

## Abstract

Pyroclastic density currents (PDCs) can generate co-PDC plumes, which segregate and buoyantly rise from the underlying gravity current. Using the atmospheric-dispersion model NAME we perform a series of co-PDC simulations that vary the particle release height and mass eruption rate for eight different weather patterns that characterise the UK and the surrounding European area. We examine the ash cloud concentration as a function of vertical elevation (flight level) within the atmosphere. We find that the ash clouds are compact in shape and often contain high (above 10 mg m^−3^) ash concentrations in the first few hours after particle release. Our results are discussed in terms of the hazard to aviation and operational modelling by volcanic ash advisory centres.

## Introduction

Fast-moving volcanic gravity currents, termed pyroclastic density currents (PDCs), can be formed by the collapse of an eruption column^1–8^, from collapse of lava domes or flow fronts^9–15^. The generated mixtures are multiphase and comprise both hot gas and solid particles, e.g. volcanic ash, lithic fragments. Accompanying plumes, commonly termed co-PDCs, and also known as phoenix clouds or co-ignimbrites^15–22^, can form from PDCs by air entrainment. The upper part of the PDC buoyantly lifts off and forms the co-PDC^18,19,21,23–25^. Therefore, co-PDCs are composed of hot gas and fine particles (the heavier particles remain in the denser, ground-hugging current). The co-PDC plumes rise until they reach a level of neutral buoyancy in the atmosphere and then disperse laterally. Given these formation conditions, co-PDCs have some unique eruption source parameters (ESPs) compared to typical vent-derived plumes, for example, co-PDCs have been found to have fine particle sizes (<90 μm)^15,18^ and a high-aspect ratio source geometry (i.e. irregular shaped, not a circular or point-source). Co-PDCs can be generated from the entire length of the underlying PDC or from a discrete part^18^ and thus do not necessarily form at the vent. Co-PDCs have the potential to occur during any explosive eruption, and their formation processes and associated ash dispersion have been observed in several historical eruptions^18^. Despite this prevalence, little work has been done on forecasting the presence and dispersal of co-PDC ash in the atmosphere.

Volcanic ash can have a significant impact on infrastructure, human health, livestock, soil fertility, and crops^2,18,26^. Ash also represents a significant hazard to aviation^27–37^, with a key example being the 2010 Eyjafjallajökull eruption, which dispersed ash over Europe, leading to an approximate US$ 2 billion loss for the aviation industry^29^. Volcanic ash (i.e. particles ≤ 2 mm) travel long distances, and disperse over wide areas^27,38^. Therefore, aircraft are not only exposed to hazard from volcanic ash close to the eruptive source, but many thousands of kilometres downwind. High ash concentrations can lead to aircraft damage, as ash entering the engines may be heated to above their glass transition temperatures^39^. This can create engine disturbance by clogging air bleed holes or sticking to surfaces^29,35,37,40^ and damage thermal barrier coatings^41–43^. Ash ingestion can also cause malfunction of electronic components or pressure losses^29,40^. Examples of impacted electronic components include speed indicators, pressure sensors, engine power, and interference with communication and navigation systems. Ash also has abrasive effects, especially on the leading edges, fan blades, or on the windshields, and this damage may only be visible after long-term exposure^29,35,37,40,44,45^.

Volcanic Ash Advisory Centers (VAAC) are responsible for providing ash hazard information to civil aviation. For the aviation industry to mitigate against the ash hazard and retain flight safety, they need to know the location of the ash cloud in the atmosphere and its ash concentration. VAACs communicate this hazard through the issuance of volcanic ash advisories^46^. In November 2025, VAACs started to issue Quantitative Volcanic Ash (QVA) information. QVA forecasts contain ranges and thresholds of ash concentration. ‘Low’ concentrations are defined as ash concentrations within the range 0.2–2 mg m^−3^. ‘Medium’ concentration is defined as 2–5 mg m^−3^ and ‘high’ ash concentration is 5–10 mg m^−3^, while ash concentrations of ≥ 10 mg m^−3^ are classified as ‘very high’^47^. The minimum satellite detection threshold of ash particles is approximately 0.2 g m^−2^ total column mass loading^29,47–50^.

**Fig. 1:**
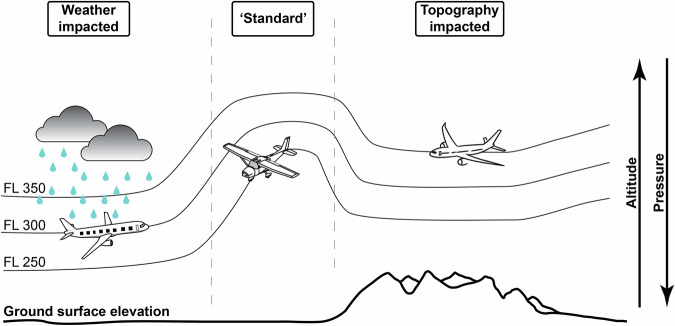
Flight levels (FL) retain vertical separation despite atmospheric pressure variations due to different weather conditions and large changes in ground topography. The 'standard' region, shown in the middle of the figure, is shown as a reference. Despite the cruise flight level being fixed (e.g. FL 300), the aircraft changes its actual altitude (i.e. km above sea level) during flight.

The International Civil Aviation Organisation (ICAO) has standard regulations on flight levels (abbreviated as FL) to provide adequate vertical separation between aircraft and sufficient terrain clearance^51^. FLs are parallel surface levels of constant atmospheric pressure, with reference to a pressure datum, FL0, which is 1013.2 hPa (1013.2 millibars) in the ‘ICAO Standard Atmosphere’^51,52^, and equivalent to mean sea level. The relationship between flight level, atmospheric pressure, and weather is shown schematically in Fig. 1. Meteorological conditions impact the altitude (above sea level) of the flight levels, for example, precipitation, often caused by a depression, lowers the atmospheric pressure and therefore the altitude of the corresponding FL. Similarly, the atmospheric pressure can be impacted by changes in the topography.

Commercial aircraft typically fly at a cruising altitude just below the tropopause^29^ between 8.8 and 12.5 km above sea level (asl)^53^, corresponding to approximately FL290–FL410 at standard atmospheric pressure. Such flights follow a set of well-defined flight phases: taxi, take-off, en-route (climb, cruise, and descent), approach, and landing^54^. A plane’s altimeter specifies the current flight altitude, and the altimeter scale corresponds to a particular barometric pressure at a certain location and time^51^. During take-off, landing, and below a certain transition altitude (a specific altitude above a minimum distance from the ground or aerodrome^51^), commonly at 457 m (1500 ft), planes set their altimeter to the current air pressure of the airport or the applied location. However, at higher altitudes (above the specific transition altitude and when in cruise flight), a standard reference set of ‘flight levels’ is used, and flying heights are determined based on pressure. This ensures that every plane uses the same reference for altitude determination for consistency. Planes do not fly at a constant altitude above the ground but rather follow an assigned flight level. For this reason, volcanic ash concentrations in the atmosphere and forecast model outputs are commonly reported per FL, conforming to regulatory (i.e. QVA) requirements.

**Fig. 2:**
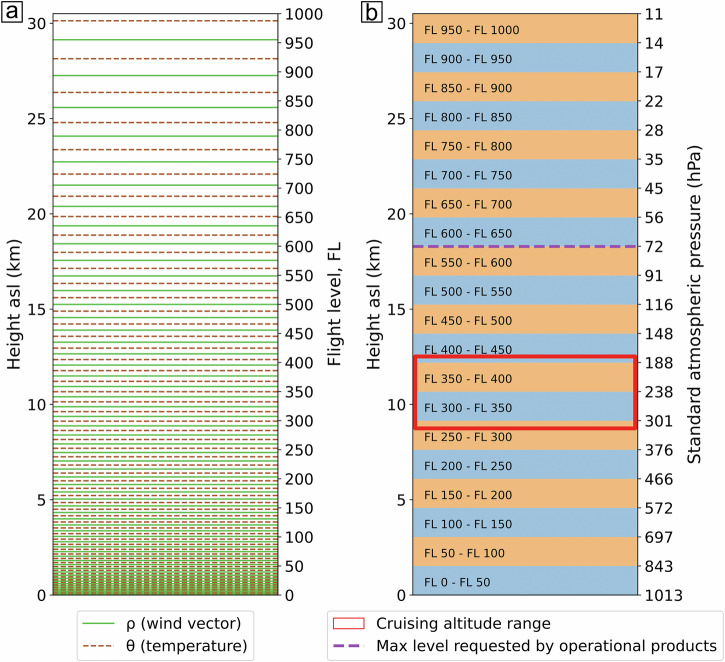
The vertical resolution of the Met Office Unified Model (MetUM) compared with flight level, FL. **a** '*ρ*' and '*θ*' levels contain the wind vector data and the temperature data, respectively. The vertical resolution of both data sets decreases with increasing height above sea level (asl). **b** NAME outputs FL in steps of 50 in this study. Despite FL being pressure-based, the standard atmosphere pressure curve is used for comparison (1013.2 hPa ≡ FL0). The purple dashed line indicates the maximum FL (FL600) requested by aviation for QVA analysis.

The UK Met Office’s Numerical Atmospheric-dispersion Modelling Environment (NAME) models particle transport and dispersion in the atmosphere by releasing a large number of model particles into a model environment. Simulations are then driven using pre-processed global atmospheric conditions from the Met Office’s Unified Model (MetUM)^55,56^, a Numerical Weather Prediction model (NWP). NAME is used by the London VAAC as its operational model for volcanic ash forecasts. Our research uses the NAME model with output results as a function of FL. The vertical resolution of both the wind vector (*ρ*) and temperature (*θ*) data reduces with height, respectively (Fig. 2). For altitudes corresponding to where commercial planes fly and the maximum FL requested by QVA (at standard atmospheric pressure: FL600), at least two *ρ* and *θ* datasets are available for each FL (at standard pressure). Whereas at altitudes >≈25 km asl, these meteorological datasets (*ρ* and *θ*) have a vertical spacing greater than 50 FL, and as such, meteorological data availability is limited.

A few studies have modelled co-PDC rise, transport, and dispersion^14,21–23,25,57–61^. Of most relevance here Hagenbourger et al.^57^ used NAME to study the sensitivity of total column mass loadings to the unique eruption source parameters (ESPs), i.e. the source area and aspect ratio, associated with co-PDCs. However, little is known about the vertical concentration of ash (in mg m^−3^) in a dispersing co-PDC cloud—which is critical for assessing the aviation hazard. While numerous publications discuss the vertical distribution of ash in vent-derived ash clouds (e.g.^19,24,62–64^), no such studies specifically focus on co-PDCs. Furthermore, given the unique ESPs of co-PDCs, limited insight can be gained from our knowledge of vent-derived plumes.

Here, we bridge this gap by (1) performing a series of NAME model runs for co-PDC ash dispersion and transport, (2) analysing the resultant atmospheric ash concentrations in the vertical (i.e. at different FLs), and (3) discussing whether the concentration could have a meaningful impact on aviation. Here, the term ‘cloud’ is used to refer to downwind ash transport and dispersion, while the term ‘plume’ solely describes the near-source behaviour.

## Methods

### Modelling and NAME setup

NAME can be used to predict atmospheric transport, dispersion, and deposition of gases and particles^65^. It uses the advection-diffusion equation and outputs the concentration of particles. Here, NAME III (v8.6)^55,65^ was used in the Lagrangian configuration to model the ash transport and dispersion of a volcanic ash cloud derived from a co-PDC plume. We used the NAME wet and dry deposition schemes^32,66–68^ and assumed ESP time homogeneity. This is an idealisation for modelling purposes, and we are aware that PDCs and co-PDCs are not constant in time (e.g. the 1980 Mount St. Helens eruption, USA^69,70^ (hereafter MSH), and the 2015 Calbuco eruption, Chile^71^).

We used the pressure-as-height coordinate system to relate the height, *z*_*ICAO*_, to the ICAO standard atmosphere pressure. Adapted from NAME^65^ this is expressed as:

1 $${z}_{ICAO=}\left\{\begin{array}{ll}\frac{{T}_{msl}}{{\gamma }_{0-11}}\left(1-{\left(\frac{p}{{p}_{msl}}\right)}^{\frac{R{\gamma }_{0-11}}{g}}\right) & {\mathrm{for}}\,p\ge {p}_{11}\\ {z}_{11}+\frac{R{T}_{11}}{g}{\mathrm{ln}}\left(\frac{{p}_{11}}{p}\right) & {\mathrm{for}}\,{p}_{11} > p > {p}_{20}\\ {z}_{20}+\frac{{T}_{20}}{{\gamma }_{20+}}\left(1-{\left(\frac{p}{{p}_{20}}\right)}^{\frac{R{\gamma }_{20+}}{g}}\right) & {\mathrm{for}}\,p\le {p}_{20}\end{array}\right.$$

with

2 $${p}_{11}={p}_{msl}{\left(1-\frac{{\gamma }_{0-11}}{{T}_{msl}}{z}_{11}\right)}^{\frac{g}{R\gamma }}$$

3 $${p}_{20}={p}_{11}\exp \left(-\frac{g}{R{T}_{11}}\left({z}_{20}-{z}_{11}\right)\right)$$

where *R* is the specific gas constant for dry air, *R* = 287.05 J kg^−1^ K^−1^; *T*_*msl*_ is the temperature at mean sea level (msl), *T*_*msl*_ = 288.15 K; *T*_11_ is the temperature at 11 km asl, *T*_11_ = 216.65 K; *T*_20_ is the temperature at 20 km asl, *T*_20_ = 216.65 K; *γ*_0−11_ is the lapse rate from 0 to 11 km asl, *γ*_0−11_ = 0.0065 K m^−1^ and *γ*_20+_ is the lapse rate at altitudes greater than 20 km asl, *γ*_20+_ = −0.001 K m^−1^. As in the ICAO standard atmosphere, the lapse rate is assumed to be constant between specified altitudes^72^. *p*_*msl*_ is the standard pressure at msl, *p*_*msl*_ = 101 325 Pa; *z*_11_ stands for the altitude of 11 km above msl, when using the hydrostatic assumption; *p*_11_ corresponds to the pressure at *z*_11_.

The applied meteorological data archive uses interpolated meteorological data in time and space (when using the Lagrangian approach)^32^. For the eruption setup, we used a pre-processed, configured NWP dataset from the global configuration of the MetUM^55,56,73,74^. The meteorology definition applied here has a global horizontal resolution with grid lengths of approximately 10 km at mid-latitudes. The computational grid in NAME has a horizontal grid resolution of 0.1° in Latitude and Longitude, and throughout the model domain, the vertical grid resolution decreases with vertical height (Fig. 2). The vertical ash concentration was output over vertical depths of 5000 ft (approximately 1500 m), which corresponds to a depth of 50 FL (at standard atmospheric pressure; Fig. 2).

Weather patterns group characteristic recurring circulation types, i.e. similar weather occurrences over a defined region. The prevalent weather pattern over the specific region can vary daily. Neal et al.^75^ defined a set of eight weather patterns for the North Atlantic and European region (30° W–20° E and 35°–70° N), which are used for seasonal and long-range forecasts and for identifying key changes in wind flow^75^. These eight weather patterns, illustrated in Fig. S1, are numbered in order of the annual historical occurrence between 1850 and 2003. The lower numbers indicate a greater frequency, i.e. over the whole year, weather pattern 1 occurs more often than weather pattern 8. For a full description, the reader is referred to Neal et al.^75^.

We used these weather patterns to consider the impact of meteorology on our results. We did not perform a climatology study, but used weather patterns to better underpin and confirm that our findings are not exclusively based on one sampled day. For each weather pattern, three different days from different seasons were manually selected for model runs. In total, we performed model runs across 24 days (Table S1), and the distribution of these days is presented in Fig. S2.

Each of these 24 days has been run for particle emission start times of 06:00 UTC, 14:00 UTC and 22:00 UTC for a forecast duration of 36 h (288 runs in total). Additionally, for 31st January 2022, we also ran a fourth time of 09:00 UTC in order to compare with the previous co-PDC study in Hagenbourger et al.^57^. The different start times were chosen by considering the diurnal cycle and the atmospheric boundary layer. The diurnal cycle refers to a daily cycle of weather changes (i.e. temperature fluctuations), and the atmospheric boundary layer (i.e. the layer between the planetary surface and free atmosphere) depends on the temperature of the ground. The lowest temperature occurs close to sunrise, between 6 and 7 a.m. local time, while the maximum occurs at 2–3 p.m. local time^76,77^. The time difference between the extremes is about ~8 h, which aligns to 8 h spacing in our run start times.

### Eruption conditions

This study used a 10 min particle release or emission time, *t*_*r*_, which corresponds to observations of large co-PDC plumes^69^. Model particles were released into the atmosphere at the level of neutral buoyancy, *z*, over a defined thickness, *dz*. This thickness of the release around *z* is given by the ratio of the total column height, *H*_*T*_, defined as^78–81^:

4 $$dz=0.3\cdot {H}_{T}.$$

Here, we approximate *dz* as ranging from 0.7⋅*H*_*T*_ to *H*_*T*_ while neglecting any overshooting top. The May 18th 1980, eruption of MSH is the largest observed and well-documented co-PDC in the literature. Therefore, in this work, we used parameter values (i.e. *A*, *α*, *dx*, *dy*, *t*_*r*_; refer to Table 1) related to this eruption. As it represents one of the largest co-PDC plumes, any reported ash concentrations and cloud footprints represent a reasonable upper limit. Exceeding these parameters, although possible, is rarely observed in nature, and such events might be governed by different physical processes. Hagenbourger et al.^57^ showed that the particle release height significantly affects the horizontal ash cloud transport, whereas the effects of source area and source aspect ratio are negligible. Following this study, here, we consider the vertical downwind concentrations of volcanic ash from a co-PDC release and its sensitivity to plume height and mass eruption rate (MER). The particles were assumed to be spherical with a total grain size distribution (TGSD) as in Hagenbourger et al.^57^ with a modal grain size in the grain size range of 26–74 μm. We applied the entire TGSD, and no distal fine ash fraction, as the entire distribution is relatively fine (i.e. 86% <105 μm). Hekla volcano in Iceland (63.98° N, 19.67° W)^82^ was used as the source location; however, the source location is not unique for our study, as we are not studying local topographic influences or volcano properties. We selected Hekla for its distance of 51 km to the Atlantic Ocean, so that the entire source area of the co-PDC remained on land.

**Table 1:** Co-PDC source parameters used in this study

Parameter	Symbol	Unit	Value(s)
Release area	*A*	km^2^	619
Aspect ratio	*α*	–	1.7
Width of source in plan view	*dx*	km	32.4
Length of source in plan view	*dy*	km	19.1
Source perimeter	*P*	km	103.0
Maximum plume height	*H*_*T*_	km	15, 20, 25 & 27
Thickness of ash release	*dz*	km	4.5, 6.0, 7.5 & 8.1
Particle emission time	*t*_*r*_	min	10
Particle density	*ρ*	kg m^−3^	2500

*A*, *α*, *dx*, *dy*, *P* and *t*_*r*_ refer to the May 18th 1980, eruption of MSH^57^. See Hagenbourger et al.^57^ for references to *H*_*T*_ and *dz*. *t*_*r*_ has been taken from Sparks et al.^69^.

The total height of co-PDC plumes has been observed^57^ in nature in the range of 1–30 km. Traditionally, *H*_*T*_ is coupled to the MER by an empirical power law^80,81,83–85^. Here, we used the MER–*H*_*T*_-relationship from Aubry et al.^83^:

5 $$\mathrm{MER}=\root{{0.226}}\of{\frac{{H}_{T}}{0.345}}.$$

The *H*_*T*_ and MER of MSH fall within the confidence interval of this relationship in Equation (5), thus applicable here, for our modelled co-PDC plumes, and used in the absence of any other specific MER–*H*_*T*_-relationship for co-PDCs. Data for very small co-PDC are not included in this relationship, we therefore limit our modelled range of *H*_*T*_ to 15 km, 20 km, 25 km, and 27 km. The aspect ratio, *α*, of the particle release area/source was set as a constant parameter describing the relationship of the source area’s width by length (*dx*/*dy*) to be 1.7 (Table 1; Hagenbourger et al.^57^).

### Numerical experiment

The volcanic ash cloud was simulated for 36 h with data output every 1 h. Table 2 defines all parameters and their variation for our set of model runs, or ‘numerical experiment’. The different values of *H*_*T*_ define unique values of *dz* and MER, following Eqs. (4) and (5), respectively. We used a particle density of 2500 kg m^−3^ and assumed no particle aggregation. All other eruption source parameters used in this study were kept constant, as defined in Table 1. The numerical experiment was then repeated for different weather patterns and dates (Figure S2).

**Table 2:** Source parameters used in the numerical experiment

Tot. plume height, *H*_*T*_ (km agl)	*dz* (km)	FL range of release region	MER (kg s^−1^)	Calculated released mass into atmosphere (kg)	Calculated released volume (m^3^)	Release start time (UTC)		
15	4.5	270–420	1.77 × 10^7^	1.06 × 10^10^	4.26 × 10^6^	06:00	14:00	22:00
20	6.0	360–560	6.34 × 10^7^	3.80 × 10^10^	1.52 × 10^7^			
25	7.5	450–670	1.70 × 10^8^	1.02 × 10^11^	4.08 × 10^7^			
27	8.1	490–750	2.39 × 10^8^	1.43 × 10^11^	5.74 × 10^7^			

Four release heights, *H*_*T*_, were used for each of the three release start times. The range of covered flight levels of the release region is indicated and rounded to the nearest ten.

The specific model run with a *H*_*T*_ = 27 km, corresponding^83^ to MER = 2.39 × 10^8^ kg s^−1^, yields a total mass release of 1.43 × 10^11^ kg for *t*_*r*_ = 10 min. This is consistent with published literature^69,84^ on MSH and thus supports our selection of source parameters. Furthermore, all our models runs can be classified as a VEI 2 on the Volcanic Explosivity Index (VEI) scale^86^. Although our plume heights are high, the released volumes from co-PDCs are small due to the short *t*_*r*_.

## Results

We focus on the impacts on the dispersed downwind cloud in terms of its location, area, concentration (with reference to QVA), and vertical mass distribution. All these results are reported across a range of weather patterns, *H*_*T*_ and MERs.

**Fig. 3:**
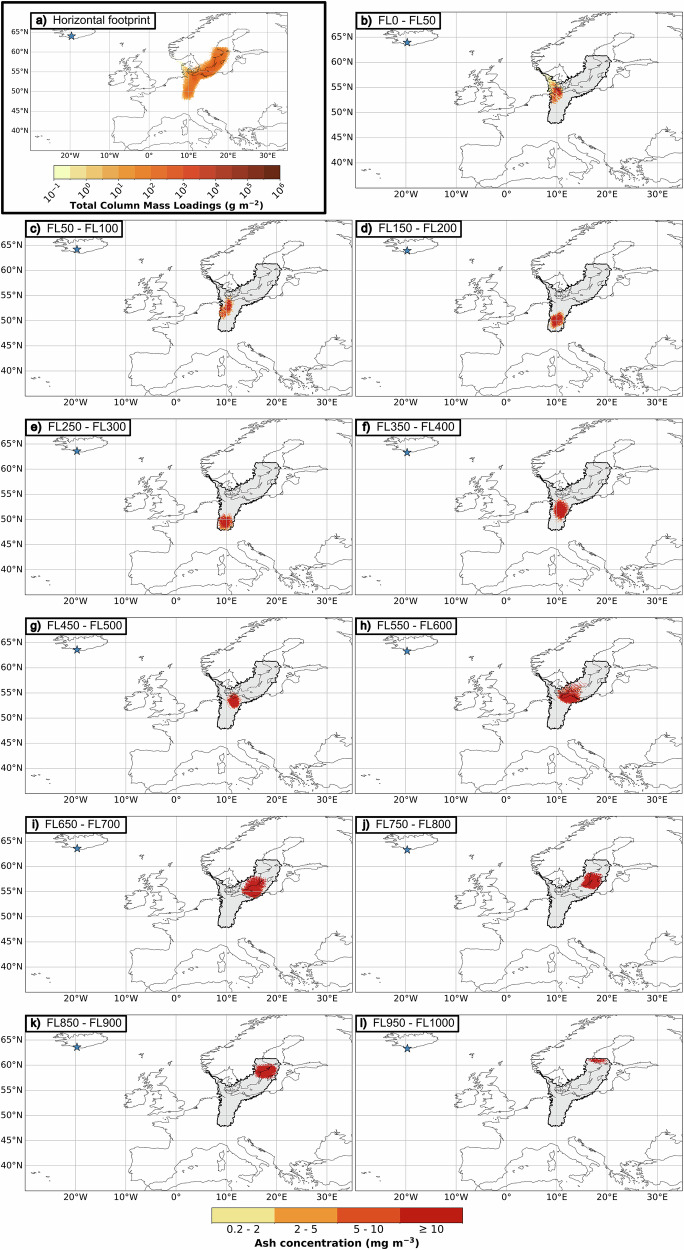
Illustration of the relationship between **a** the horizontal footprint of total column mass loadings (g m^−2^) and **b–l** the vertical output shown at selected flight levels (in mg m^−3^) at 12 h after the particle release. The black outline in (**b**–**l**) represents the boundary of total column mass loadings ≥ 0.2 g m^−2^. Particles were released at *H*_*T*_ = 27 km on 31st Jan 2022 at 14:00 UTC. In all panels, a blue star indicates the release location. Note the difference in the range and the units of the colour bars between the total column (**a**) and vertical outputs (**b**–**l**).

Figure 3 provides an illustrative example of how concentrations at specific vertical intervals (i.e. at specific FLs) relate to commonly reported total column mass loadings. The total column mass loadings (g m^−2^), shown in Fig. 3a, are the total integrated mass through the entire vertical column. Figure 3b–l represent the ash concentration (mg m^−3^) within different altitude layers (i.e. at different flight levels). The maximum extent of the ash cloud with total column mass loadings ≥ 0.2 g m^−2^, constrains the total horizontal footprint. The regions of ash located within each FL vary in size, shape, location, and ash concentration (Fig. 3b–l), but sum to provide the total column mass loading (Fig. 3a). In this example, at the highest FLs, the cloud area is relatively small, whereas for the middle FLs (i.e. FL300–FL600), the cloud varies in size and is typically more elongated in shape. The shape, area, and concentration also depend on the time since particle release.

### Ash cloud location

**Fig. 4:**
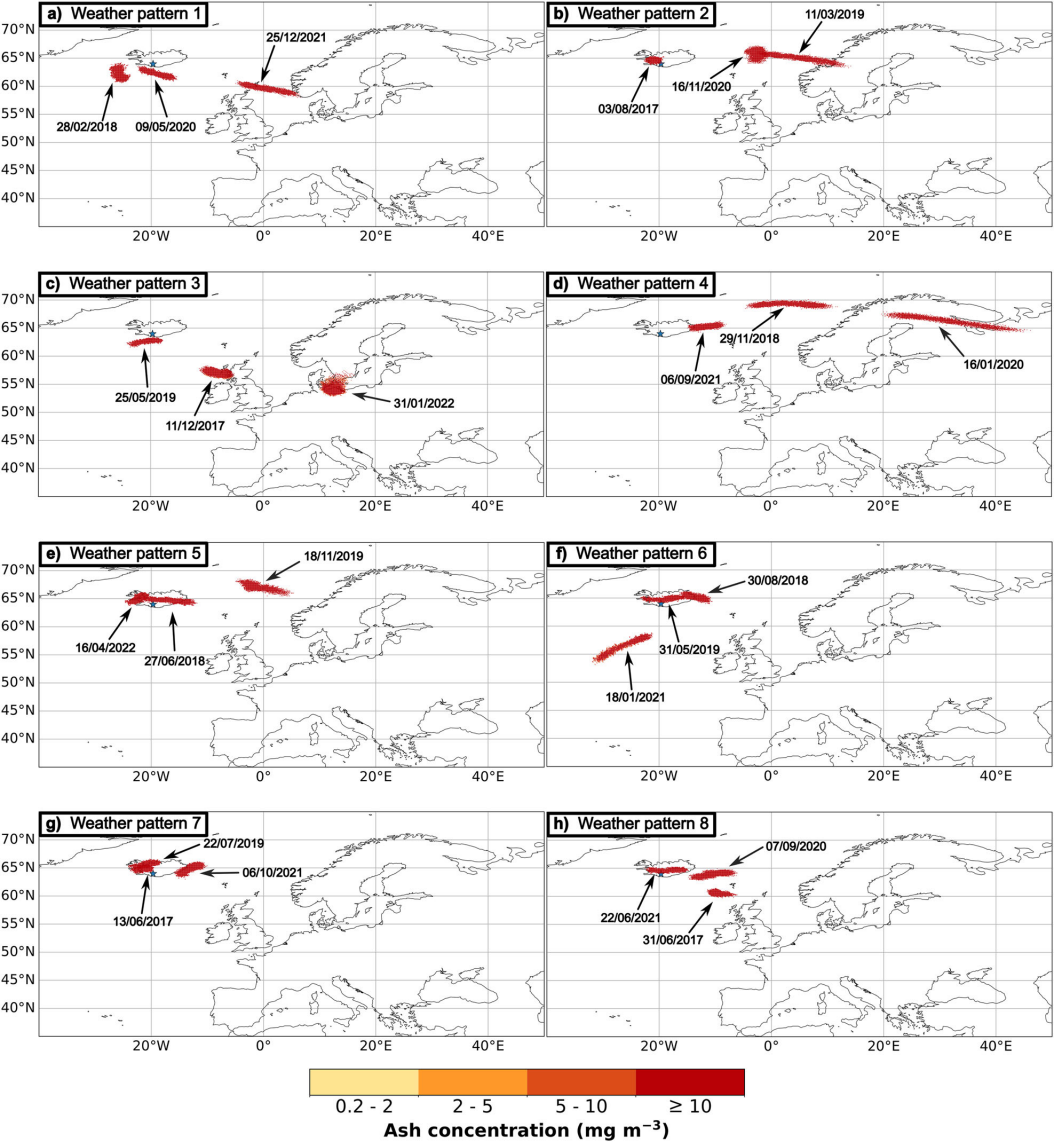
Ash cloud location displayed for the eight weather patterns at 12 h after particles were released at *H*_*T*_ = 27 km at 14:00 UTC. FL550-FL600 is displayed for all. Each subfigure (**a**–**h**) contains three separate NAME model outputs, and thus displays three different ash clouds generated for each weather pattern. These are indicated by the black arrows, and the dates correspond to the associated eruption/release start. Some of the ash clouds within a weather pattern slightly overlap in this figure. In all panels, a blue star indicates the release location, and the data are given as ash concentration in mg m^−3^.

The North Atlantic and European region can be characterised by eight weather patterns^75^ which group similar weather occurrences over a defined region. We now evaluate if the ash cloud location systemically varies between each weather pattern (Fig. 4). We have chosen to display *H*_*T*_ = 27 km because the most (vertical) diversity was shown; however, the trends described in this section remain true for all the different flight levels. Our full dataset can be found in the supplementary information (Fig. S3 displays FL300–FL350 and Fig. S4 displays FL900–FL950).

Across all of the eight weather patterns, we found that the ash clouds (Fig. 4; all at 12 h since particle release at FL550–FL600) were remarkably discrete and isolated; they feature a highly concentrated region, with very minor lower concentration margins. Throughout the whole range of weather patterns at FL550–FL600, QVA classification declares the ash concentration as ‘very high’ (≥10 mg m^−3^), as seen by the dark red coloured ash clouds in the outputs. Also, the cloud trajectories were mainly towards Europe for all runs and weather patterns. However, even within the same weather pattern at the same period of time after the particle release, the specific location of the ash cloud is highly variable, and both the cloud shape and size often show large variability.

The three ash transport and dispersion outputs corresponding to weather pattern 1 (Fig. 4a) are all located in different positions—there is no spatial overlap between the outputs. Two of the ash clouds are located relatively close to Iceland, while the other ash cloud is elongated and stretched close to the north coasts of Scotland and Norway. For weather pattern 2 (Fig. 4b), one cloud is highly elongated while the other two are more compact and remain closer to the source. Outputs for weather pattern 3 (Fig. 4c) are widely spread in terms of location; however, they exhibit a similar plume shape. In general, all of the outputs for weather pattern 4 (Fig. 4d) are elongated; however, like the outputs from weather pattern 3, they are widely spread in terms of location while considering the same point in time. Ash concentrations from weather patterns 5 and 6 (Fig. 4e, f) both show two ash clouds located directly over the Icelandic land mass, while the third is located in the Arctic Ocean and the Atlantic Ocean, respectively. For weather patterns 7 and 8 (Fig. 4g, h), the outputs illustrate that the ash clouds have not travelled far from the source within 12 h.

**Fig. 5:**
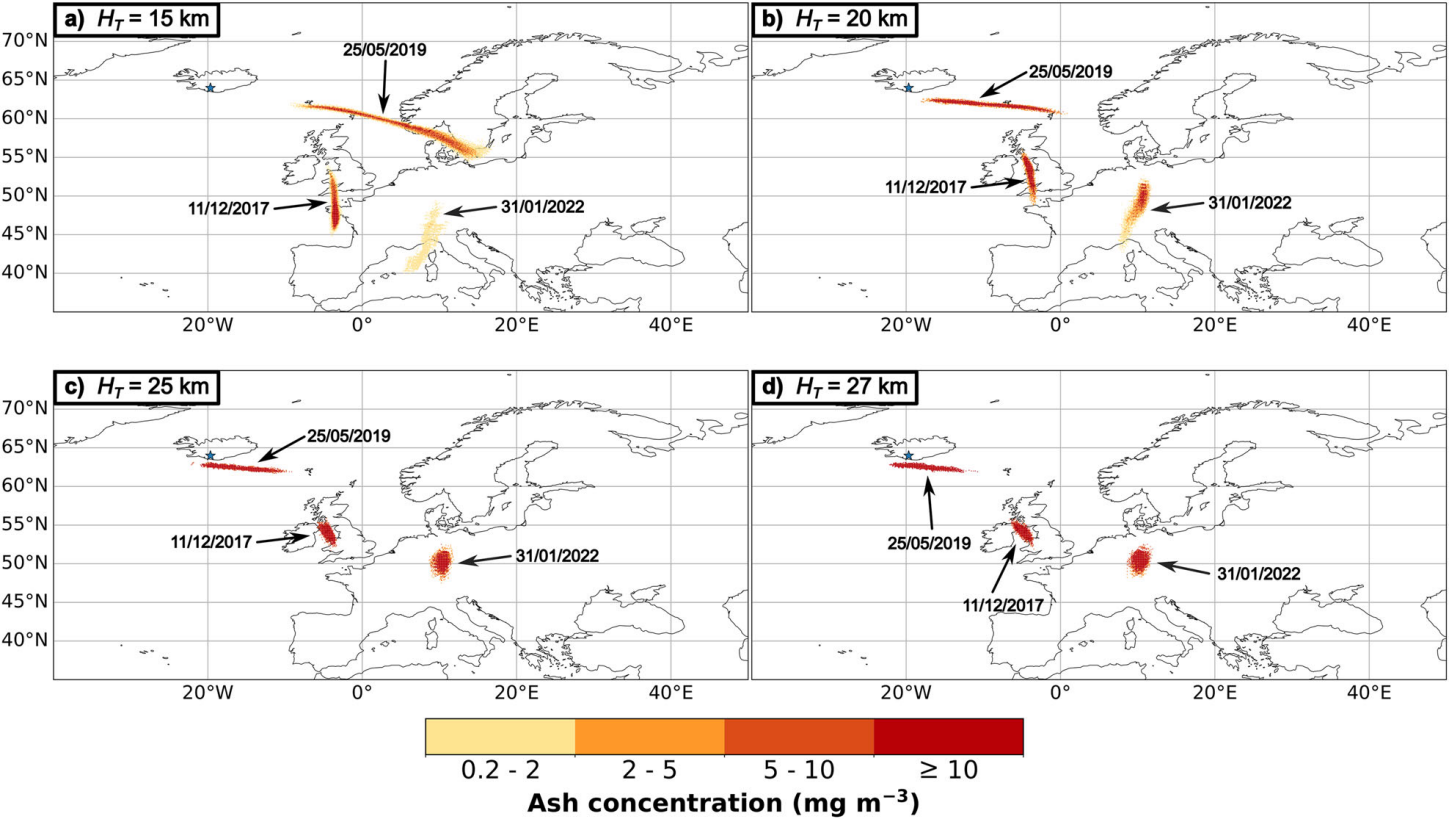
Ash cloud locations for particle release heights for weather pattern number 3 at 12 h since particle release start time. **a** *H*_*T*_ = 15 km, **b** *H*_*T*_ = 20 km, **c** *H*_*T*_ = 25 km, and **d** *H*_*T*_ = 27 km. Each subfigure shows three different model run outputs at FL300–FL350 for different dates. In all panels, a blue star indicates the release location, and the data are given as ash concentration in mg m^−3^.

Figure 5 compares the ash concentration (in mg m^−3^) as a function of *H*_*T*_ for a specific flight level (here, FL300–FL350, where commercial planes fly), and a specific weather pattern (here, number 3). For a given day, the ash cloud locations are similar for all release heights (*H*_*T*_ = 15 km, 20 km, 25 km and 27 km), simply highlighting how the ash cloud dispersal is highly sensitive to the specific meteorological conditions. However, the cloud extent, ash concentration, and shape differ as a function of *H*_*T*_. Ash released at *H*_*T*_ = 25 km and *H*_*T*_ = 27 km (Fig. 5c, d) show similar behaviour in terms of their location, size, concentration, and shape. *H*_*T*_ = 15 km (Fig. 5a) contains mainly low to medium ash concentrations (yellow and orange colours), whereas very high ash concentrations are present in both *H*_*T*_ = 25 km and *H*_*T*_ = 27 km (Fig. 5c, d) (dark red, ≥10 mg m^−3^). The location of the ash cloud at the higher FLs (e.g. FL550–FL600 and FL900–FL950) does not change significantly when *H*_*T*_ is varied (Figs. S5 and S6, respectively). However, the ash cloud location within lower FLs is more sensitive to *H*_*T*_ (Fig. 5).

### Ash cloud area at each flight level

**Fig. 6:**
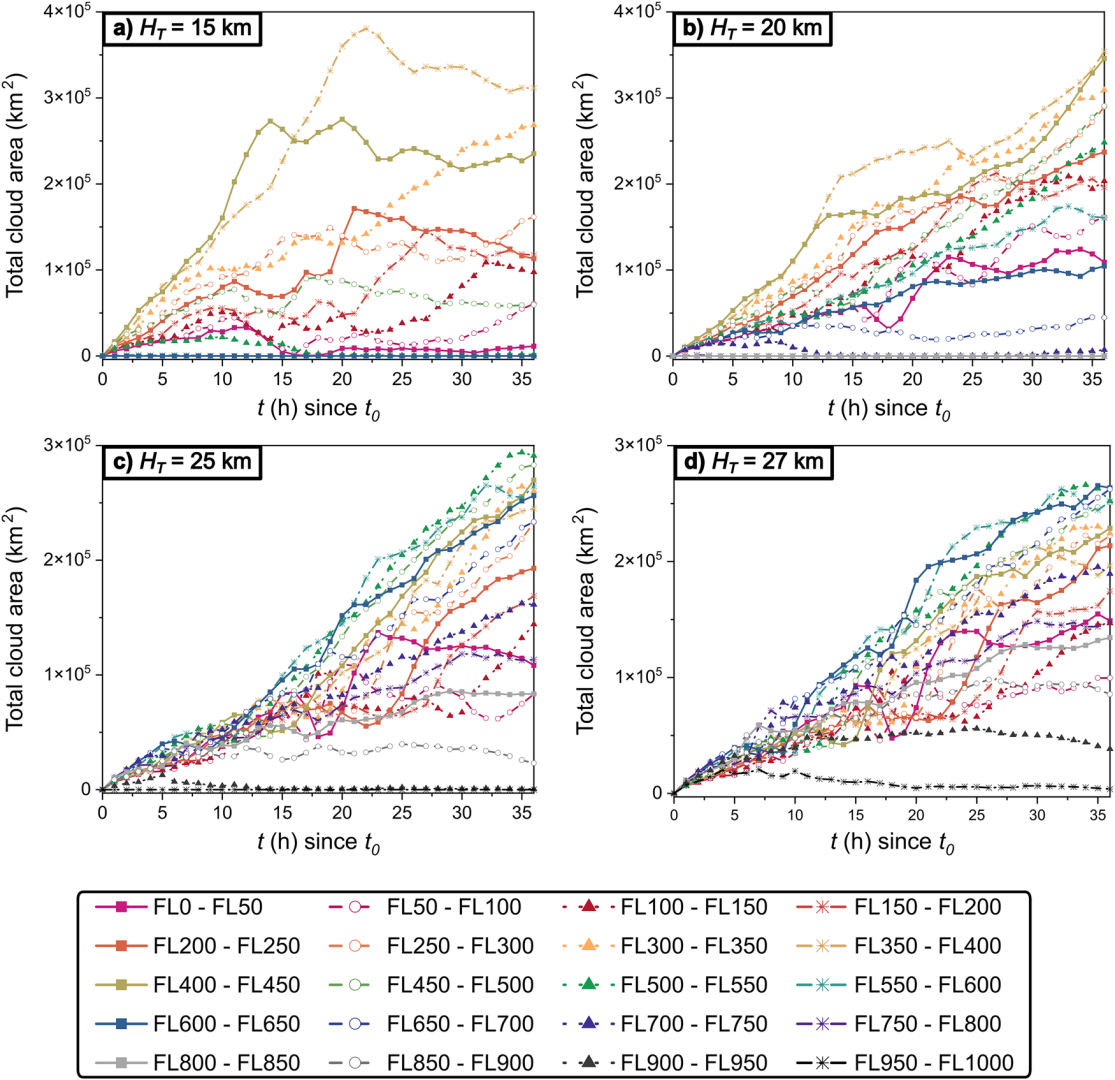
Total ash cloud area at each 50 FL as a function of time for particle release heights. **a** *H*_*T*_ = 15 km, **b** *H*_*T*_ = 20 km, **c** *H*_*T*_ = 25 km, and **d** *H*_*T*_ = 27 km. The displayed data originates from the same weather pattern (3), day (31st January 2022), and start time (14:00 UTC). We applied a lower ash concentration threshold of 0.2 mg m^−3^ to define the edge of the cloud. The lines between data points are not model fits and are just used to guide the eye. The legend specifies the colour and data marker corresponding to each FL interval.

The total area of the downwind ash cloud is reported as a function of time for each *H*_*T*_ with ash concentrations ≥ 0.2 mg m^−3^ (Fig. 6). We chose the same date (31st January 2022, 14:00 UTC) as was used to illustrate the ash cloud behaviour (Fig. 3). For all values of *H*_*T*_, divergence in total ash cloud area is observed with time. The areal extent of the cloud spreads noticeably from the time of release and shows little particle deposition, i.e. no large reductions in cloud area within the first 36 h. *H*_*T*_ = 15 km (Fig. 6a) generates an ash cloud reaching FLs up to approximately FL600; *H*_*T*_ = 20 km generates a cloud up to FL800 (purple coloured lines), *H*_*T*_ = 25 km reaches up to FL950, while *H*_*T*_ = 27 km covers the full FL range investigated here, up to FL1000. Figure 6c, d, corresponding to *H*_*T*_ = 25 km and *H*_*T*_ = 27 km, respectively, show similar cloud area distributions across the different flight levels. Across all release heights, the range of FL250–FL500, corresponding to the orange, yellow, and green lines in Fig. 6, is among the largest cloud areas for all time steps analysed. Furthermore, although the absolute magnitude of these co-PDC ash cloud areas is relatively small (~300,000 km^2^), the main altitude range they occupy corresponds to flight levels used for commercial aircraft (FL300–FL400).

**Fig. 7:**
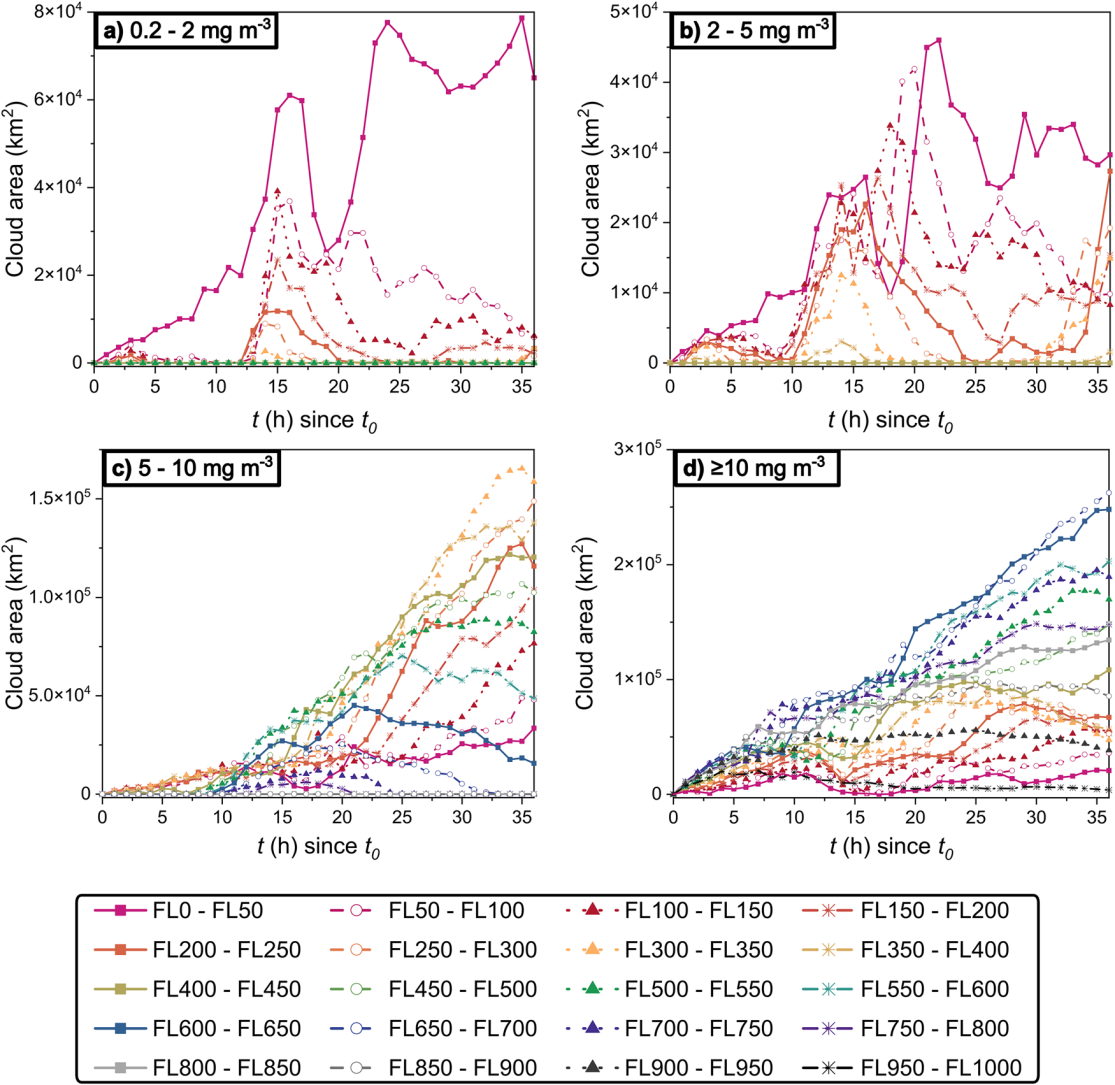
Ash cloud area within each 50 FL interval as a function of time for particle released at *H*_*T*_ = 27 km. The different subplots correspond to the different QVA thresholds **a** 0.2–2 mg m^−3^, **b** 2–5 mg m^−3^, **c** 5–10 mg m^−3^, and **d** ≥10 mg m^−3^. The data originate from the same day (31st January 2022) and start time (14:00 UTC). The lines between data points are not model fits and are just used to guide the eye. The *y*-axis varies in scale between the panels. The legend specifies the colour and data marker corresponding to each FL interval.

We can also analyse the ash cloud area corresponding to each of the different QVA thresholds. As an illustrative example, Fig. 7 shows the ash cloud areas within the QVA threshold ranges for *H*_*T*_ = 27 km and particle release on 31st January 2022, 14:00 UTC. For completeness, all the other release heights are provided in the supplementary information (Fig. S5–S7). This analysis confirms that for *H*_*T*_ = 27 km and *H*_*T*_ = 25 km the generated clouds have mostly ‘high’ (5–10 mg m^−3^) to ‘very high’ (≥10 mg m^−3^) ash concentrations across the entire set of FLs, and FLs ≥ FL500 only contain ash concentrations ≥ 5 mg m^−3^ (Figs. 7c, d and S9c, d). Furthermore, for *H*_*T*_ ≥ 20 km, most of the cloud area corresponding to the 0.2–2 mg m^−3^ concentration range is located near to the ground level (pink and orange coloured lines), which reflects particle settling (Figs. S8d, S9d and 7d). In contrast, for *H*_*T*_ = 15 km, the lowest *H*_*T*_ studied, most of the cloud contains lower ash concentrations 0.2–2 mg m^−3^ (Fig. S7a), and ash concentrations with 10 mg m^−3^ are not present in most FLs after 27 h since particle release (Fig. S7d). However, FL350–FL450 (covering commercial airspace use) are still impacted by ‘very high’ ash concentrations throughout the 34 h modelled for.

### Vertical location of the maximum mass

**Fig. 8:**
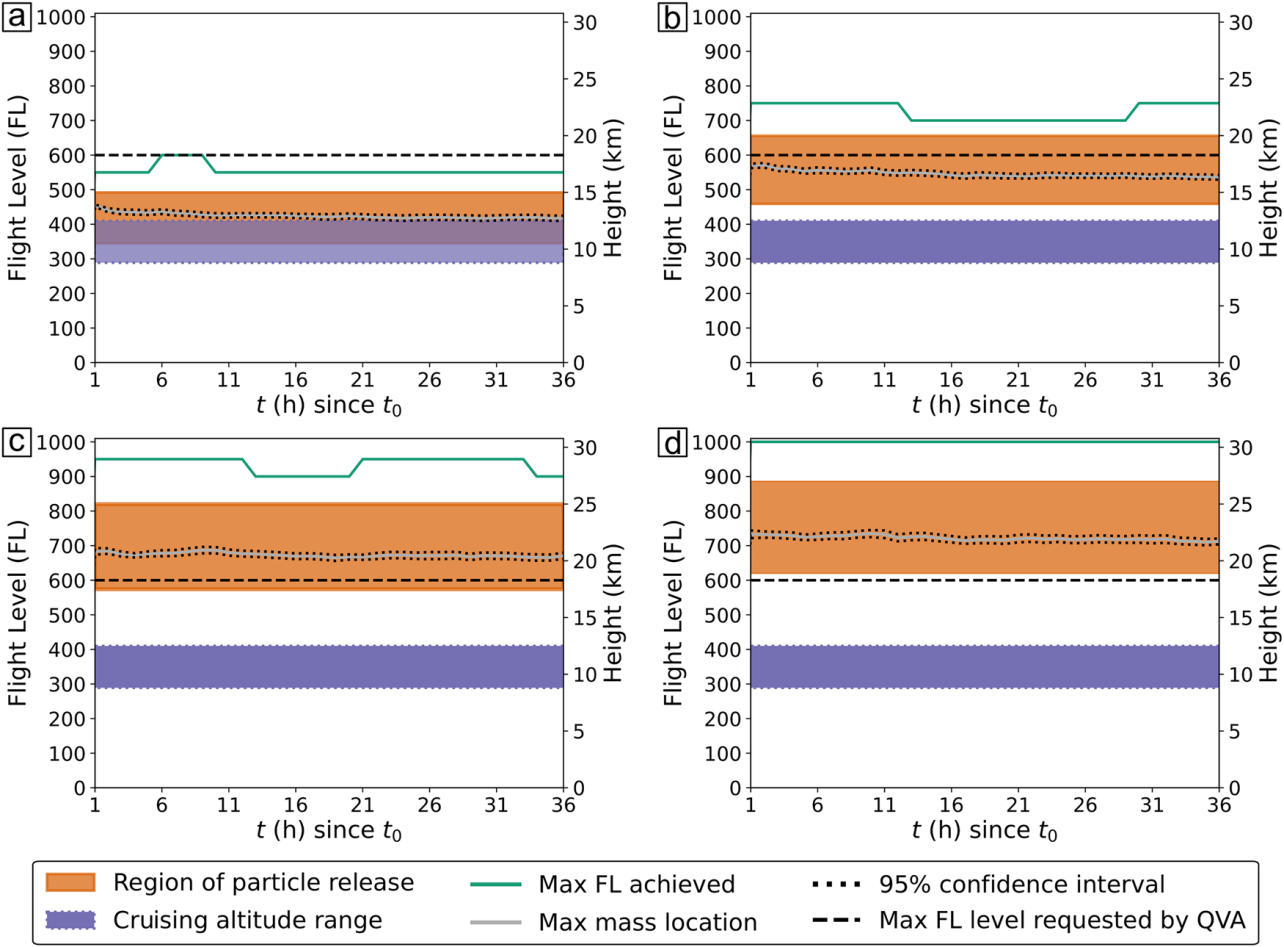
Maximum mass of particles in the atmosphere as a function of time for particle release heights of **a** *H*_*T*_ = 15 km, **b** *H*_*T*_ = 20 km, **c** *H*_*T*_ = 25 km, and **d** *H*_*T*_ = 27 km. The orange boxes correspond to the particle release regions, the purple boxes refer to the range of commercial aircraft cruising altitudes^53^, ~FL290 to FL410. The green lines indicate the average maximum FL achieved by particles for all model runs. The light grey lines indicate the average FL location of the maximum mass, and the black dotted lines represent the 95% confidence interval, including all weather patterns, days, and release start times. The black dashed lines represent the maximum requested FL to be reported in QVA products.

It is also important for aviation to consider where the dispersed ash resides in terms of mass. Here, this is done by reporting the vertical location where (i) the maximum amount of ash is located and (ii) the highest FL that the ash reaches for concentrations ≥ 0.2 mg m^−3^. The minimum ash cloud height is simply taken as ground level, as ash settles from the cloud. For all start times across all weather patterns, the maximum FL achieved by the co-PDC ash is always situated above the release region (orange box in Fig. 8) by approximately 50–150 FLs (Fig. 8). The small fluctuations in time are likely to originate from vertical atmospheric turbulence. The light grey line indicates the average location of the maximum amount of mass, which decreases with time but remains, at least for the first 36 h, within the original release region. The 95% confidence interval (black dotted lines) of these data shows that there is very little variation in these results for different days (weather patterns) and start times.

We note that the computational domain of NAME in the vertical direction in our experiments is restricted to 30 km (approximately corresponding to FL1000). The maximum FL achieved for *H*_*T*_ = 27 km shown in Fig. 8d, reaches the top of the computational domain, and it is therefore possible that some particles may be able to reach higher altitudes.

## Discussion

Our results and findings are a consequence of the unique ESPs for co-PDCs, and here, we discuss our results in the context of the aviation hazard. Firstly, we discuss the influence of weather pattern and release height. We found that the shape, size, or location characteristics of the co-PDC ash clouds cannot be related to a specific weather pattern. Even if the weather pattern is known, we also cannot predict the vertical cloud location (i.e. at a specific flight level). For a given weather pattern, the distance travelled by the ash cloud (up to 12 h) can also vary significantly. The only generalisation that can be made is that weather patterns 5–8 (Fig. 4e–h) result in less dispersed ash clouds and remain closer to the source location over Iceland, relative to weather patterns 1–4 (Fig. 4a–d). This means that, based on our current knowledge, robust statements and generalisations about how the location, area, or shape of an ash cloud from a co-PDC vary as a function of the weather pattern cannot be made. A model simulation using the specific weather data for the event is required to obtain the specific characteristics of the ash cloud shape, size, or location. Future work should compare our findings with the whole set of 30 weather patterns defined by Neal et al.^75^. Any generalisations linking dispersion and transport characteristics to weather patterns are more likely with a larger data set of weather patterns. For example, Harrison et al.^87^ are able to identify mean, characteristic flow speeds in the mid-upper troposphere for each weather pattern when the full set of 30 is used. Here, in our case, we suggest that the relatively similar behaviour of ash clouds dispersed in weather patterns 5–8 (Fig. 4e–h) originates from the grouping method, as together they only contain 9 of the 30 weather patterns, while weather patterns 1–4 (Fig. 4a–d) contain 21 out of 30 weather patterns (Fig. S1).

Previous work^57^ has confirmed that the plume/release height is an extremely important ESP for modelling co-PDC ash dispersal, just like vent-derived eruptions^66,88^. Here, we have shown that changes in the ash concentration also depend on the *H*_*T*_ and associated MER, where only high (*H*_*T*_ = 25 km and 27 km) release heights result in high ash concentrations (Fig. 5c, d) in the first few hours since emission. As previously detailed, we modelled the co-PDC plumes as an ash release at a specific height range and not as a line source from the vent up to the plume top (as is done operationally by the London VAAC for standard vent-derived plumes). This impacts our observations as the particles are not released into the entire atmosphere but rather only into the stratosphere. The short *t*_*r*_ (10 min) is also of importance, as all the ash particles experience the same wind fields (including wind shear effects) and are therefore likely to remain in a similar location, with concentration changes largely due to different MERs, and total erupted mass.

For *H*_*T*_ = 25 km and *H*_*T*_ = 27 km, we observe very similar cloud behaviour in terms of location, shape, size, extent, and concentration (i.e. Fig. 5). They are also similar in terms of the total cloud area distribution (i.e. Fig. 6c, d). This is due to the high altitude in the stratosphere, where the particles are released. At these altitudes, less turbulence acts on the ash particles^89^.

Our results highlight the importance of knowing *H*_*T*_ and any variations thereof during an ongoing eruption. For example, variation in a (vent-derived) *H*_*T*_ of 6 km by a 1 km uncertainty can impact the estimated minimum and maximum cloud area by a factor^62^ of 3. Given the dispersion model output varies greatly with *H*_*T*_ and thus MER for both co-PDC plumes and vent-derived plumes^55,57,62,83,90,91^, it would be beneficial to the forecasts and operational setups to know the exact release height from co-PDC plumes during a real-time event to initialise the dispersion modelling. This includes reconciling data and observations from different measurement methods such as visual ground-based observations, pictures or video footage, different remote sensing techniques (ground-based radar, lidar, (visible) cameras, and satellites or aircraft observations), and potentially coupling them with numerical models^27,29,30,46,90–101^. Furthermore, these observations can be hindered by meteorological cloud coverage, adverse weather, differentiation between ash and ice or water, and night-time light limitations^18,27,29,102,103^. Finally, although the relationship between *H*_*T*_ and MER (Eq. (5)) has been shown to hold for large co-PDC events, the limits of this MER–*H*_*T*_ relationship remain to be tested for the full range of co-PDC plume heights.

The newly introduced QVA concentration thresholds help evaluate risk of volcanic ash encounters for the aviation industry. We have shown that the cloud extent with ash concentrations ≥ 0.2 mg m^−3^ increases with time (for at least the first 36 h). For short times after particle release, we observe high-concentration ash clouds with small to non-existent areas of lower ash concentrations at the cloud margins (0.2–5 mg m^−3^; e.g. Figs. 3–5). Across all our model runs, the maximum ash concentration achieved was 28,840 mg m^−3^ (modelled for 3rd August 2017 at 1 h after particle release, *H*_*T*_ = 27 km at FL650–FL700). However, similar high ash concentrations are observed throughout the whole dataset (often ≥ 10 mg m^−3^) and are thus a common feature of the co-PDC clouds modelled.

Previous work^33^ has modelled the maximum ash concentration range generated from the 26th February 2000 eruption of Hekla, Iceland, along the flight path of a NASA DC-8 research aircraft that encountered ash on the 28th February 2000 and suffered engine damage^33,104^. The Hekla eruption column^33,105^ with heights up to 12 km, reached a maximum ash concentration of 4–5 mg m^−3^ on the flight path. Similarly, Witham et al.^33^ modelled the ash concentrations during the 24th June 1982 eruption of Galunggung, Indonesia. An ash encounter with a British Airways Boeing 747 aircraft led to failure of all four engines^29,33^ and the modelled concentrations reached a maximum of ~45–320 mg m^−3^ for plume heights of 12–16 km, with large uncertainties given the uncertain eruption column height^33^. More broadly, the maximum ash concentration modelled by Witham et al.^33^ for five different volcanic ash aircraft encounters is 200 mg m^−3^. For each simulation, they used a power law relationship between *H*_*T*_ and MER, which compares well with the Mastin^84^*-H*_*T*_-MER relationship. Furthermore, previous work^62^ has analysed the ash concentration of the 2010 Eyjafjallajökull eruption on 6th May and found forecasted peak ash concentrations of 13 mg m^−3^ at FL0–FL200. This is consistent with the 13 mg m^−3^ peak ash concentration found on 7th May by Beckett et al.^55^ in FL0–FL200 and FL200–FL350 across different model parameter settings. For the determination of MER, both studies^55,62^ used the Mastin^84^-*H*_*T*_-MER relationship and applied a 5% distal fine ash fraction scaling, which accounts for physical processes such as aggregation and fall-out of large, heavy particles close to the source.

For our co-PDC clouds, we found the maximum ash concentration to be 28,840 mg m^−3^ located at FL650–FL700 for *H*_*T*_ = 27 km, which corresponds to a MER of 2.4 × 10^8^ kg s^−1^. At FL300–FL400 (corresponding to commercial airspace use), for the same model run and time since release start, the ash concentrations are ≤ 721 mg m^−3^. This concentration is in line with previous NAME studies of ash-aircraft encounters from vent-derived plumes. However, the maximum ash concentrations found within our co-PDC clouds are extremely high relative to those previously reported for vent-derived plumes. This can be explained well by our different modelling approach, which reflects, at least in part, the different co-PDC source conditions. Firstly, we only released the particles in the top 30% of the plume (refer to Equation (4)) to reflect transport and dispersion at a level of neutral buoyancy. Secondly, we used a fine TGSD appropriate for co-PDCs and have not applied a distal fine ash fraction scaling, meaning that the entire mass is applied to the downwind dispersion. Thirdly, we used higher *H*_*T*_ and thus MERs compared to these previous studies on vent-derived plumes.

We now consider the mass distribution within the ash cloud (Fig. 8). After a short *t*_*r*_ (10 min), we track the mass of ash within the cloud as it moves downwind. Within the first 36 h, across all weather patterns, dates, and start times, we observe two consistent behaviours. (i) The maximum FL achieved remains 50–150 flight levels above the release region within the first 36 h. (ii) The elevation of maximum mass sits within the release region. The fine co-PDC grain size^15,18^ contributes to this observation, as these particles have long atmospheric residence times and can travel far distances^18,27,38^. Particle sedimentation is still present, but the settling velocity is low compared to the vertical component of air velocity^106^. These findings are useful for future operational setups at VAACs that wish to consider the ash concentration from a co-PDC. The elevation of maximum mass is only expected to align with FLs used for commercial airspace when release heights are smaller than *H*_*T*_ = 20 km. However, as previously shown, the ash concentration remains high (relative to QVA) across most FLs (for release at ≥ *H*_*T*_ = 20 km).

The different eruption start times provide both different weather conditions and boundary layer levels^76,77^, but no extreme differences or patterns in the resulting ash cloud are observed. The focus on vertical atmospheric layers, e.g. flight levels, means that convective and vertical mixing needs to be considered, and the way these are modelled within NAME might have a minor impact on the vertical location, the particles achieve. Future work could continue to test these results by examining different release locations outside of Iceland and by further varying the meteorological impact. For example, previous observations at different latitudes found that the volcanic plume height attained is impacted by humidity and wind shear^96,107^.

Our results also have potential implications for other dispersion events. In this study, we used the TGSD documented for the Campanian co-ignimbrite eruption^108^. This TGSD has a modal grain size of 37 μm and is likely to be representative of other co-PDC plumes^18^, as all co-PDCs form by a common self-selecting process wherein segregated particles buoyantly lift off. However, this remains to be verified by further field and real-time investigations. Additionally, this work could be expanded and compared to other events that (re)suspend particulate matter in a similar way. This could include resuspended ash events^21,27,109–114^, for example, as they show a similar grain size characteristics to co-PDCs^109,111^. Where the grain size modes of remobilised ash have been shown^111^ to be 32–63 μm. Additionally, sub-Saharan dust^115–117^ has a similar grain size^117^ with a mode 1–30 μm.

To conclude, here, we studied the vertical ash concentration, transport, and dispersion of co-PDC ash clouds from the ground surface (FL0) up to FL1000 (~30 km altitude) using eruption source parameters that are appropriate for (large) co-PDCs. We focused on large co-PDCs to provide an upper limit on the likely natural range. We showed that within our dataset, the ash cloud location, shape, or size at each 50 flight levels cannot be directly related to a specific weather pattern, and thus these cloud characteristics cannot be pre-determined. We also observed ‘very high’ ash concentrations (≥10 mg m^−3^) for all release/plume heights above *H*_*T*_ = 20 km. The ash clouds were observed to be compact in shape with little to no reduction in concentration towards the cloud margins. For all release heights under study (*H*_*T*_ = 15 km, 20 km, 25 km, and 27 km), variations in the ash concentration at each FL interval were observed. However, little variation in the associated cloud location is observed between the different FLs for a given plume height at 12 h after particle release. *H*_*T*_ = 25 km and *H*_*T*_ = 27 km generated clouds display a similar location and shape, due to the ash being released into the stratosphere. The total cloud area exceeding 0.2 mg m^−3^ at each 50 FL interval increases with time (within the first 36 h) and hence increases the hazard to aviation. Again, within the first 36 h, the elevation of the maximum mass resides within the release region, and the maximum flight level achieved by the ash is 50–150 flight levels above the release region. Although the ash clouds generated from co-PDCs have a small total area (typically <300,000 km^2^), they comprise very high ash concentrations (max. 28,840 mg m^−3^) and thus, in the event of an eruption producing a co-PDC plume, could pose a significant hazard to aviation.

## Supplementary information

Supplementary Information

## Data Availability

NAME III Version 8.6 was used in the transport and dispersion model simulations. The UK Met Office NAME model and UM output to drive NAME are available via license from the UK Met Office (©Crown Copyright, Met Office): https://www.metoffice.gov.uk/research/approach/modelling-systems/dispersion-model. The data-generating script used in this contribution can be found on Zenodo (Hagenbourger et al.^118^).
