# Supplementary material for: The ash concentration of co-PDC clouds: implications for operational modelling and the aviation hazard
Source: NPJ Nat Hazards. 2026 May 7;3(1):56. doi: 10.1038/s44304-026-00214-7 (PMC13364718; doi:10.1038/s44304-026-00214-7)
Supplement: Supplementary file 1 — Supplementary Information [file 44304_2026_214_MOESM1_ESM.pdf]

npj Natural Hazards

Supporting Information for

'The ash concentration of co-PDC clouds: implications for operational modelling and the aviation hazard'

Marie Hagenbourger[1*], Thomas J. Jones[1], Frances M. Beckett[2], and Samantha L. Engwell[3]

[1] *Lancaster Environment Centre, Lancaster University, Lancaster, United Kingdom*
[2] *Met Office, Exeter, United Kingdom*
[3] *British Geological Survey, The Lyell Centre, Edinburgh, United Kingdom*
[*] *Corresponding authors. Email: m.hagenbourger@lancaster.ac.uk*

Additional Supporting Information (File uploaded to: https://doi.org/10.5281/zenodo.17611537)

.txt-File F1. Exemplary NAME input file '*NAME_SI_maininput.txt*'
This template can be used with NAME to reproduce all the data used for this publication. The specific parameters and changes are highlighted in Table 2. This specific template has been used for experimental run *a_14* of 31st January 2022 (weather pattern 3).

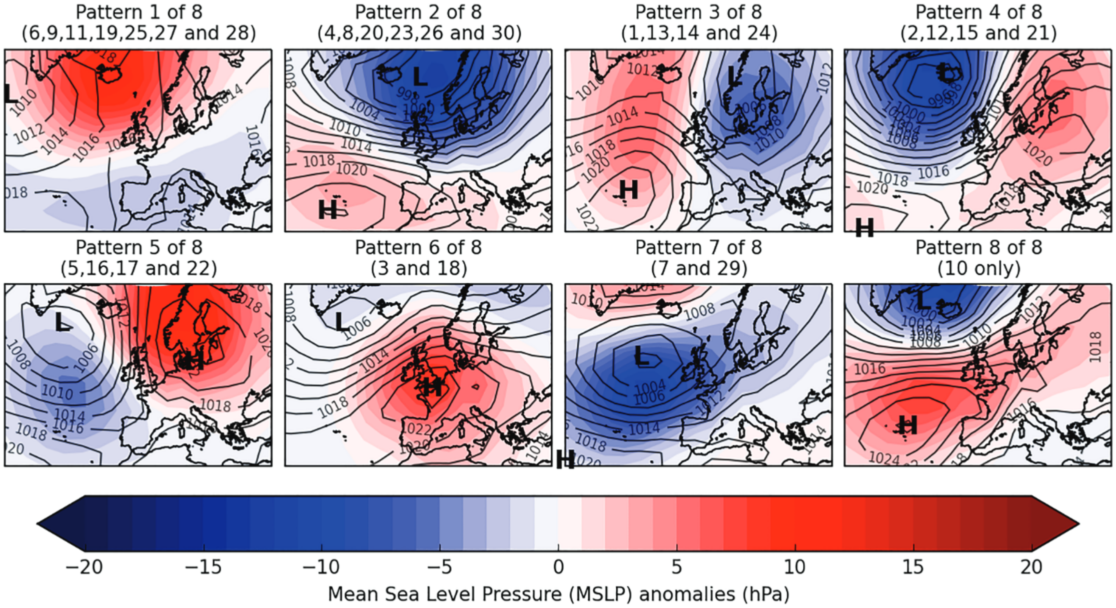

Figure S1: An overview of the eight weather patterns. A set of 30 sub-patterns used for medium-range and variation examination is grouped into their specific weather pattern. These sets are indicated in brackets at the top of each subplot. The weather patterns show the location of positive Mean Sea Level Pressure (MSLP) and negative MSLP over the UK. The MSLP is given as coloured contours in 2 hPa intervals, and the Mean Sea Level Pressure anomalies are represented by the colour bar. Figure taken from Neal *et al.*[1] with permission from Wiley.

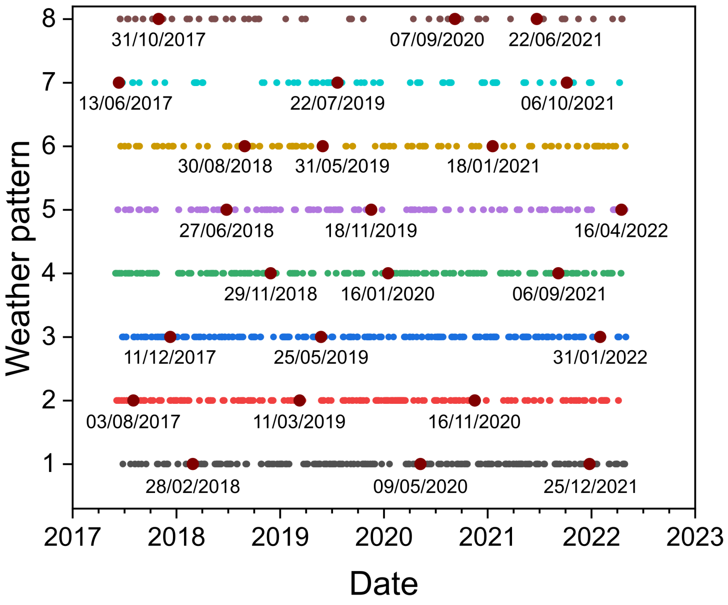

Figure S2: Visualisation of the selected days for our NAME runs. The dataset range is indicated per weather pattern and is shown by small symbols in different colours. Datasets highlighted in dark red circles are used in this study with their dates given in the format day/month/year.

Table S1: Dates manually chosen per weather pattern. Each weather pattern covers three different seasons.

Weather pattern	Day	Month	Year
1	28	2	2018
	9	5	2020
	25	12	2021
2	3	8	2017
	11	3	2019
	16	11	2020
3	11	12	2017
	25	5	2019
	31	1	2022
4	29	11	2018
	16	1	2020
	6	9	2021
5	27	6	2018
	18	11	2019
	16	4	2022
6	30	8	2018
	31	5	2019
	18	1	2021
7	13	6	2017
	22	7	2019
	6	10	2021
8	31	10	2017
	7	9	2020
	22	6	2021

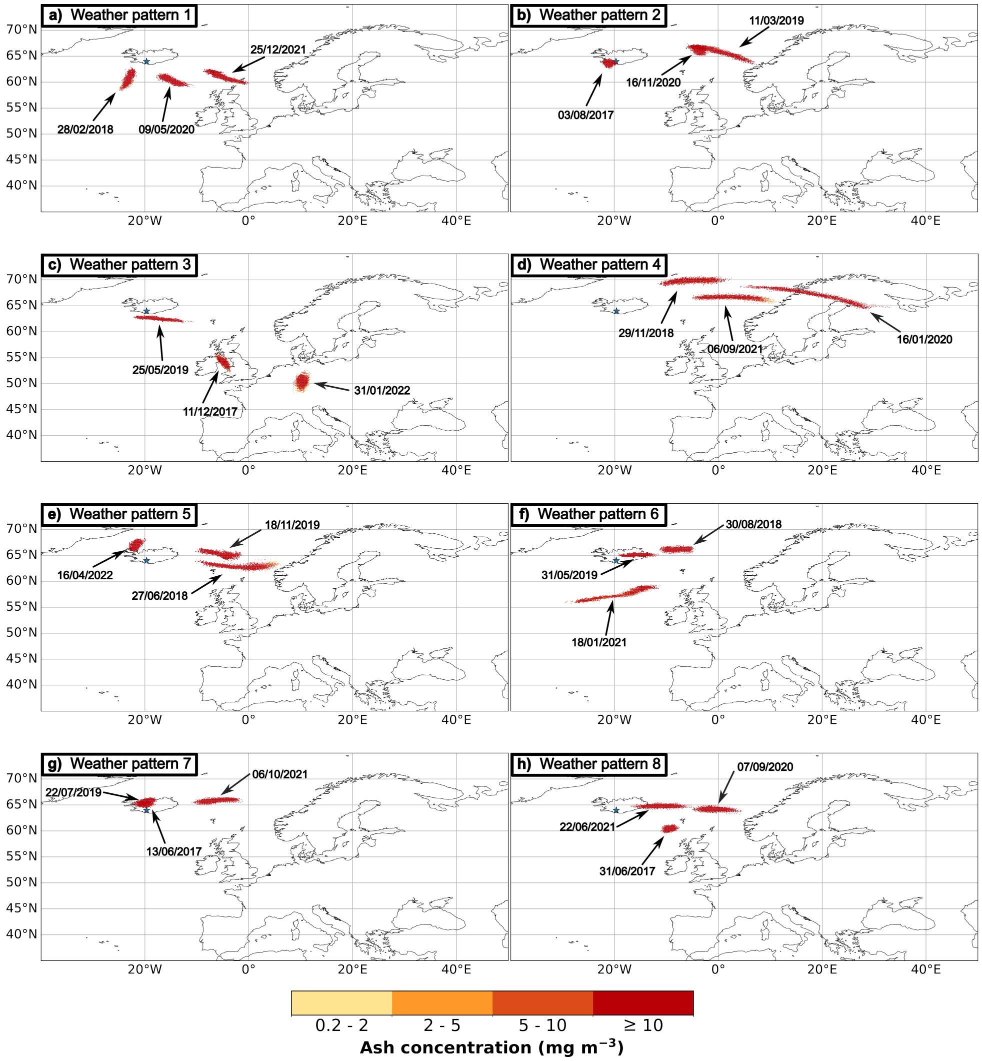

Figure S3: Ash cloud location displayed for the eight weather patterns at 12 h after particles were released at $H_T = 27\,\text{km}$ at 14:00 UTC. FL300 - FL350 (included in the commercial plane range) is displayed for all. Each subfigure contains three separate NAME model outputs, and thus three different ash clouds generated for each weather pattern. These are indicated by the black arrows, and the dates correspond to the associated eruption/release start. Some of the ash clouds within a weather pattern slightly overlap in this figure. In all panels, a blue star indicates the release location, and the data are given as ash concentration in mg m^{-3}.

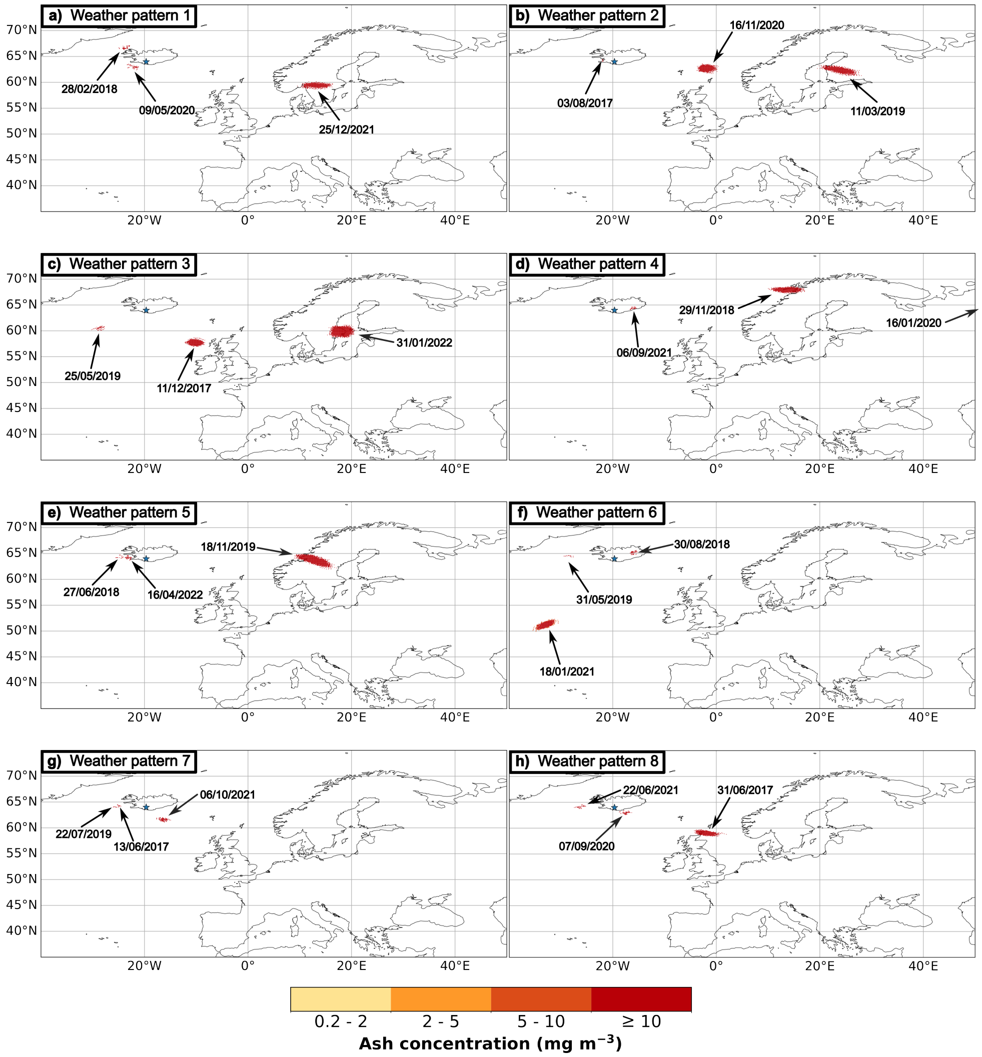

Figure S4: Ash cloud location displayed for the eight weather patterns at 12 h after particles were released at $H_T = 27$ km at 14:00 UTC. FL900 - FL950 is displayed for all. Each subfigure contains three separate NAME model outputs, and thus three different ash clouds generated for each weather pattern. These are indicated by the black arrows, and the dates correspond to the associated eruption/release start. Some of the ash clouds within a weather pattern slightly overlap in this figure. In all panels, a blue star indicates the release location, and the data are given as ash concentration in mg m^{-3}.

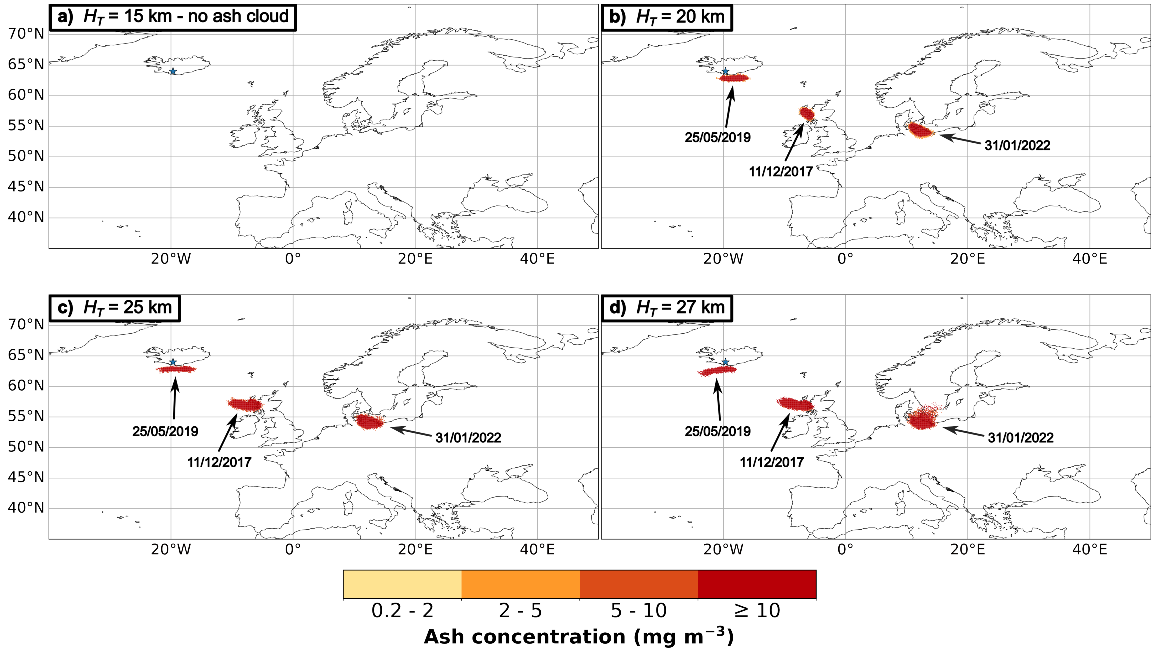

Figure S5: Ash cloud locations for particle release heights of **(a)** $H_T = 15\,\text{km}$, **(b)** $H_T = 20\,\text{km}$, **(c)** $H_T = 25\,\text{km}$, and **(d)** $H_T = 27\,\text{km}$ for weather pattern number 3 at 12 h since particle release time. Each subfigure shows three different model run outputs at FL550- FL600 for different dates. In all panels, a blue star indicates the release location, and the data are given as ash concentration in $\text{mg}\,\text{m}^{-3}$.

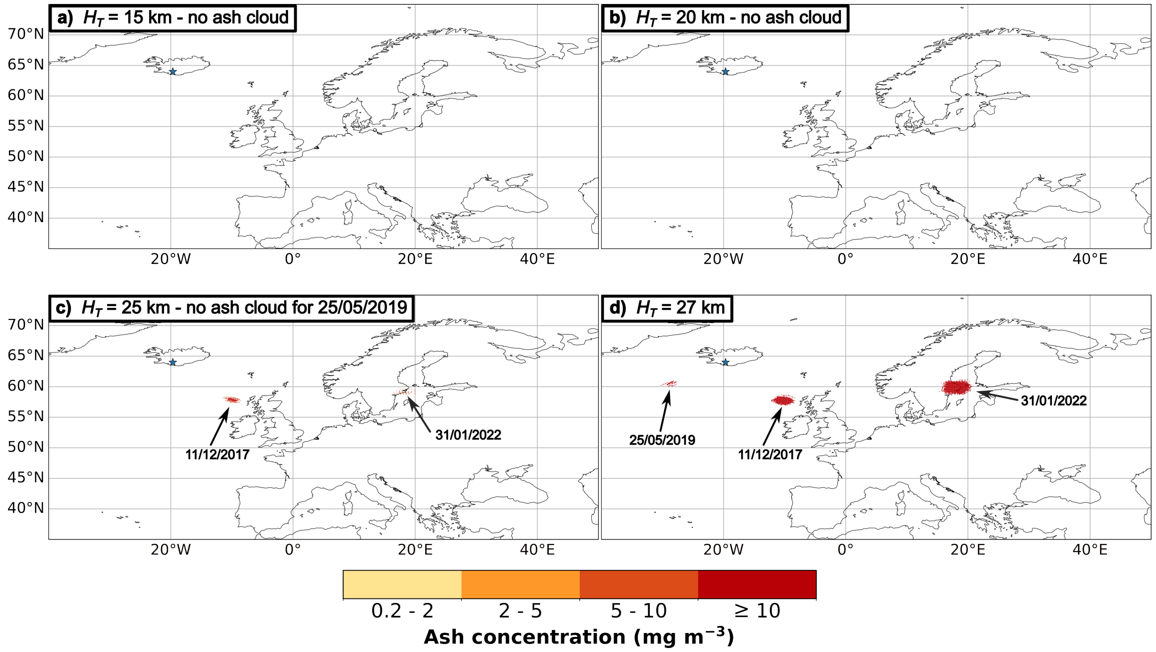

Figure S6: Ash cloud locations for particle release heights of **(a)** $H_T = 15\,\mathrm{km}$, **(b)** $H_T = 20\,\mathrm{km}$, **(c)** $H_T = 25\,\mathrm{km}$, and **(d)** $H_T = 27\,\mathrm{km}$ for weather pattern number 3 at 12 h since particle release time. Each subfigure shows three different model run outputs at FL900 - FL950 for different dates. In all panels, a blue star indicates the release location, and the data are given as ash concentration in $\mathrm{mg\,m^{-3}}$.

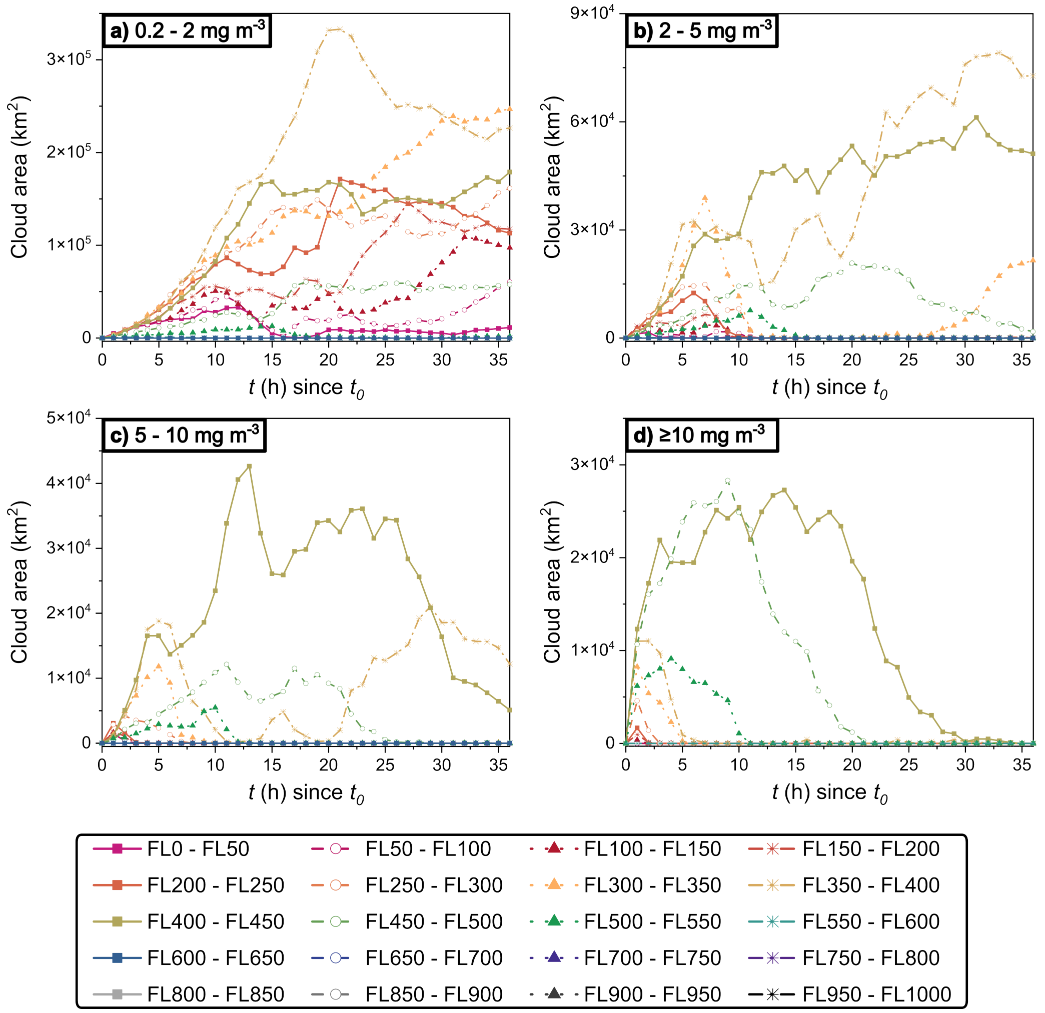

Figure S7: Ash cloud area within each 50 FL interval as a function of time for particle released at $H_T = 15\,\text{km}$. The different subplots correspond to the different QVA thresholds **(a)** 0.2-2 $\text{mg}\,\text{m}^{-3}$, **(b)** 2-5 $\text{mg}\,\text{m}^{-3}$, **(c)** 5-10 $\text{mg}\,\text{m}^{-3}$, and **(d)** $\geq 10\,\text{mg}\,\text{m}^{-3}$. The data originate from the same day (31st January 2022) and start time (14:00 UTC). The lines between data points are not model fits and are just used to guide the eye. The y-axis varies in scale between the panels. The legend specifies the colour and data marker corresponding to each FL interval.

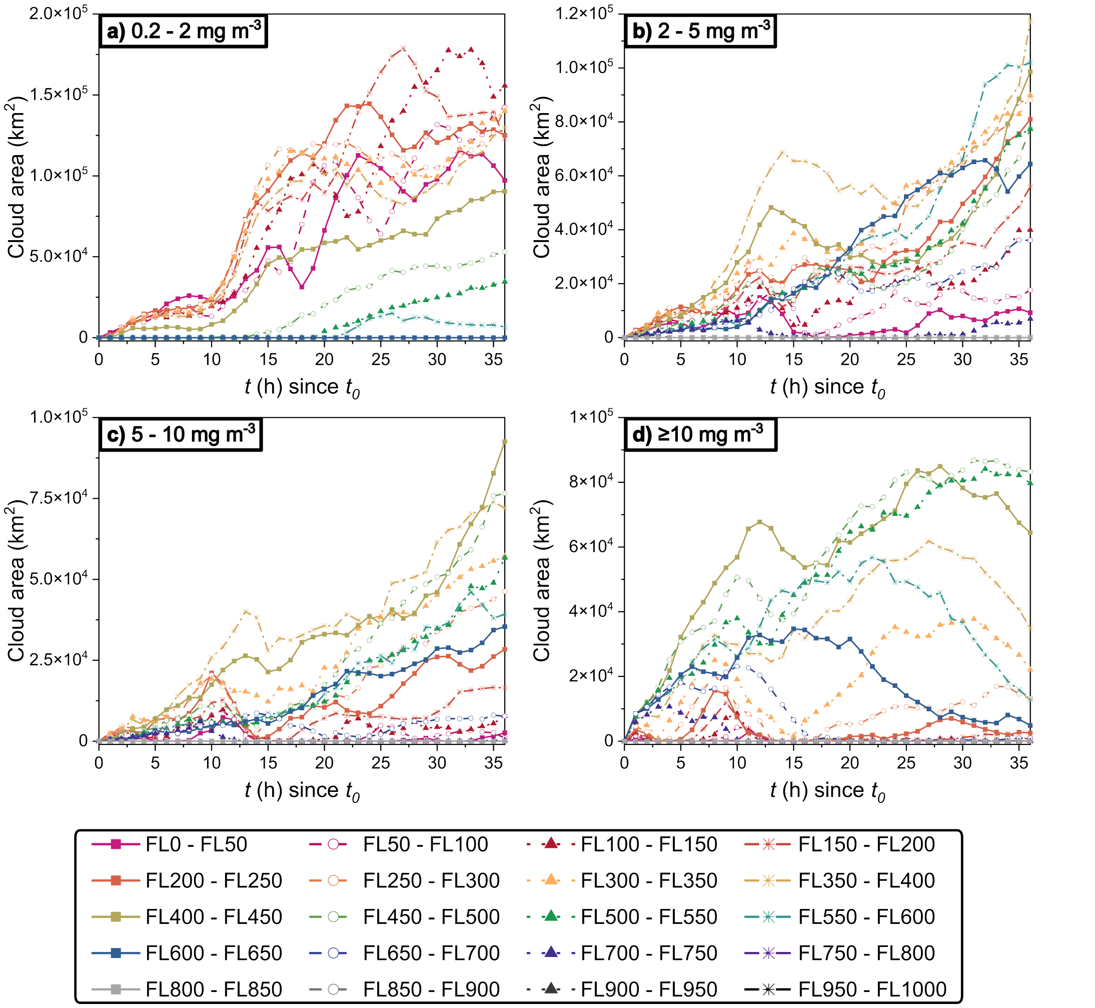

Figure S8: Ash cloud area within each 50 FL interval as a function of time for particle released at $H_T = 20\,\text{km}$. The different subplots correspond to the different QVA thresholds **(a)** 0.2-2 $\text{mg}\,\text{m}^{-3}$, **(b)** 2-5 $\text{mg}\,\text{m}^{-3}$, **(c)** 5-10 $\text{mg}\,\text{m}^{-3}$, and **(d)** $\geq 10\,\text{mg}\,\text{m}^{-3}$. The data originate from the same day (31st January 2022) and start time (14:00 UTC). The lines between data points are not model fits and are just used to guide the eye. The y-axis varies in scale between the panels. The legend specifies the colour and data marker corresponding to each FL interval.

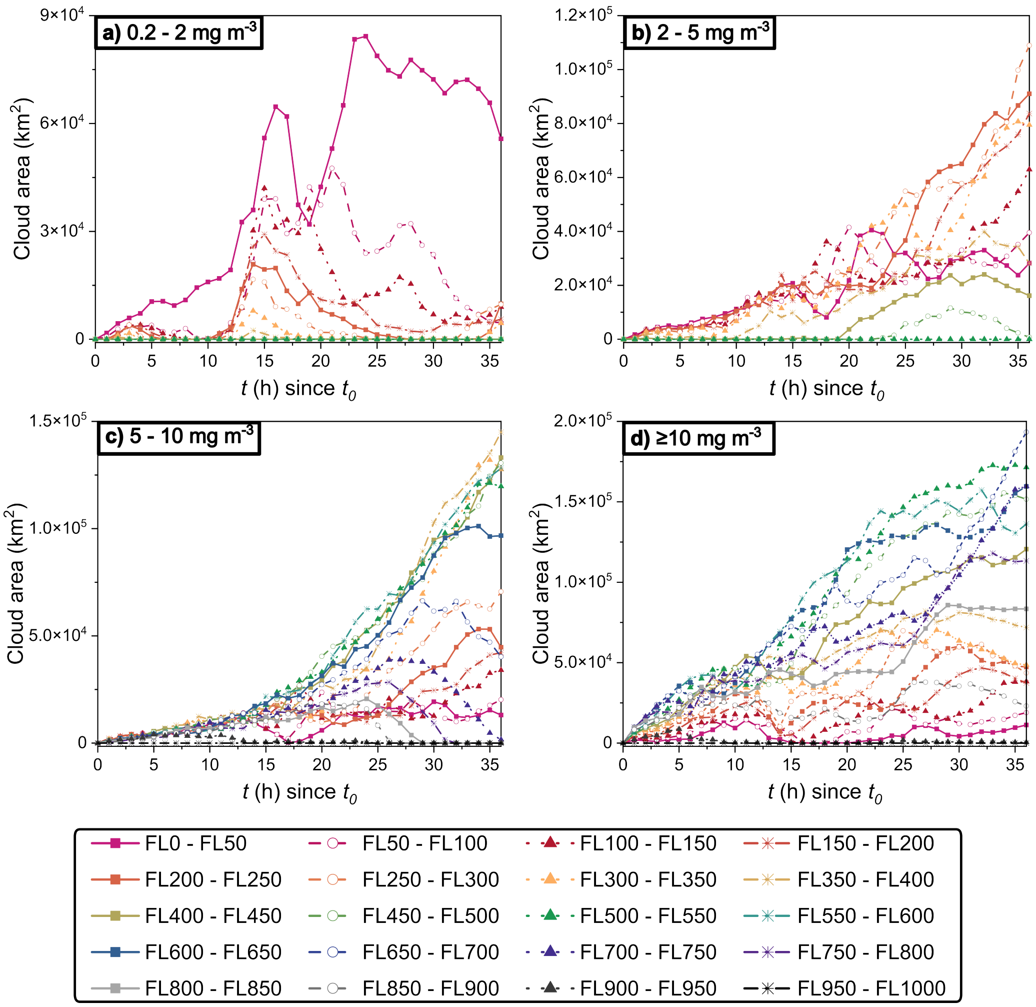

Figure S9: Ash cloud area within each 50 FL interval as a function of time for particle released at $H_T = 25\,\text{km}$. The different subplots correspond to the different QVA thresholds **(a)** 0.2-2 $\text{mg}\,\text{m}^{-3}$, **(b)** 2-5 $\text{mg}\,\text{m}^{-3}$, **(c)** 5-10 $\text{mg}\,\text{m}^{-3}$, and **(d)** $\geq 10\,\text{mg}\,\text{m}^{-3}$. The data originate from the same day (31st January 2022) and start time (14:00 UTC). The lines between data points are not model fits and are just used to guide the eye. The y-axis varies in scale between the panels. The legend specifies the colour and data marker corresponding to each FL interval.

References

1. Neal, R., Fereday, D., Crocker, R. & Comer, R. E. A flexible approach to defining weather patterns and their application in weather forecasting over Europe. en. *Meteorological Applications* **23,** 389–400. ISSN: 1469-8080. doi:10.1002/met.1563 (2016).
